# The Eyes

**Published:** 1877-05

**Authors:** 


### *THE EYES.*

No one can fail to be interested in the improvements that have been made to remedy the defects of impaired eyesight. We recently spent an hour or two most profitably at the store of Mr. I. Kahn, corner of Fourth Avenue and Tenth Street. Mr. Kahn is one of the most thorough Opticians in the country. He does not keep a store simply for the sale of glasses and spectacles, without regard to the condition of the eyes of those who are to wear them, as is usually the case with dealers in such goods, but he makes a careful and intelligent examination of the eyes by means of instruments constructed for that purpose, and selects or fits glasses or pebbles that are adapted to the requirements of the eyes in every case.

It not unfrequently happens that one eye of the same individual will be near-sighted and the other far-sighted, requiring one glass to be concave and the other convex. Mr. Kahn discovered this peculiarity in a patient we sent him, and whose sight was becoming gradually impaired by the use of glasses not suited to the condition of the eyes; one eye did nearly all the work, while the other was becoming weak from want of exercise. He said he would put the weak eye to work, and thus increase its strength by making it useful. There are, unfortunately, but few thoroughly competent opticians, and these deserve our highest appreciation. They are not often found in the large establishments; a lower order of skill and intelligence is sufficient to conduct an indiscriminate trade of glasses for money.

